# An Unusual Origin of the Left Vertebral Artery: A Case Report

**DOI:** 10.7759/cureus.43824

**Published:** 2023-08-20

**Authors:** Nagendra Singh Sonwani, Kishor S Thakur, Navneet Ateriya, Satish Verma

**Affiliations:** 1 Forensic Medicine, Pt. Jawahar Lal Nehru Memorial Medical College, Raipur, IND; 2 Forensic Medicine, Government Medical College and Hospital, Kanker, IND; 3 Forensic Medicine and Toxicology, All India Institute of Medical Sciences, Gorakhpur, IND; 4 Forensic Medicine, University College of Medical Sciences, New Delhi, IND

**Keywords:** medicolegal cases, autopsy, transverse foramen, aortic arch, vertebral artery

## Abstract

The present paper describes a 53-year-old male who presented to the emergency department with a history of sudden chest pain followed by unconsciousness at home. The autopsy revealed the left vertebral artery (LVA)'s aberrant origin from the aortic arch between the left common carotid artery and subclavian artery in a male deceased during a medicolegal autopsy.

The aortic origin of the vertebral artery is an unusual branching pattern. Knowledge about the vertebral artery's normal and variant arterial anatomy is essential for patients who undergo various surgical or interventional procedures. In the sudden death of cardiac origin, its anatomy and morphology also hold crucial value. Typically, the LVA arises from the first part of the left subclavian artery (LSA).

## Introduction

The aortic arch typically gives rise to three significant vessels, which include the brachiocephalic trunk on the right and left common carotid and the left subclavian artery (LSA) on the left side. The right vertebral artery originates from the right subclavian artery, while the left vertebral artery (LVA) originates from the LSA. It then enters the foramen of the transverse process in C6 vertebrae, passes through other foramina upward in the same plain, and finally enters the brain through the foramen magnum [1-6].

Different types of variations in LVA are noted and classified in the literature. Adachi first classified this variation into types A, B, and C based on its origin. The present case is concerned with the aberrant origin of the LVA, which is a type C variation as per the classification given by Adachi [1]. LVA arises from the fourth branch of the aortic arch, just medial to LSA. The frequency of this variation ranges between 0.68% and 5.8%. In most cases, the person remains asymptomatic unless atherosclerotic changes develop in the vessels. However, its prevertebral course is crucial as atherosclerosis frequently affects it [7,8].

The knowledge of the anatomical and morphological variation of the vertebral artery is crucial not only in clinical settings but also during autopsies. Here, we present a case of the sudden death of cardiac origin due to the blockage of coronary arteries with the uncommon aberrant origin of the LVA.

## Case presentation

A 53-year-old male experienced chest pain, sudden unresponsiveness, and loss of consciousness at home. The patient was taken to the emergency room of the tertiary care institute. After examination by the attending doctor, he was declared dead, and the body was sent to the mortuary for a medicolegal autopsy. During the external assessment, it was noted that the person was 172 cm in height and weighed 88 kg. He was well-nourished with a dark brown skin complexion. Rigor mortis had developed over the whole body. Post-mortem lividity was present over the back and fixed except for pressure points. There were no signs of mechanical trauma over the body. His face and eyes appeared to be congested. Other external findings were unremarkable.

Upon internal examination, the heart weighed 390 gm and was examined by the inflow-outflow method. The wall thickness measured 1.5 cm and 3.1 cm for the right and left ventricles, respectively. The left descending coronary artery showed 85% of the lumen's blockage by hardened yellowish atheromatous plaque, 3.5 cm from the left coronary Ostia. The right coronary artery showed 75% of the lumen's blockage by plaque, 2.9 cm from the right coronary Ostia. On further dissection, the left anterior descending artery and the left circumflex artery showed complete blockage at the level of 1.0 cm and 1.2 cm from their origin, respectively. We observed a white fibrotic patch measuring 2.8 x 1.3 located anteriorly over the left ventricle's epicardium surface.

**Figure 1:**
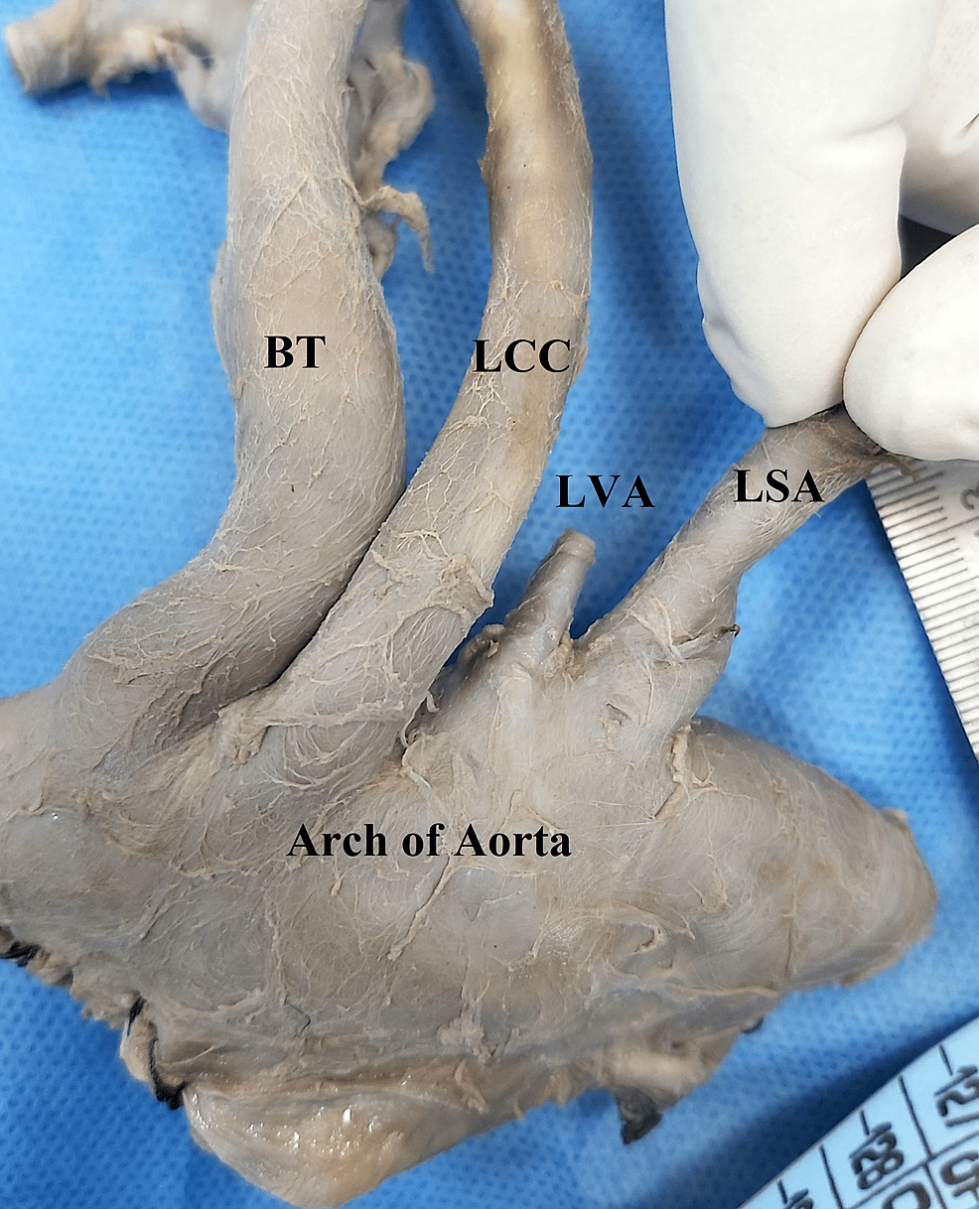
Branching pattern of vessels from the aortic arch including the anomalous origin of the LVA BT: brachiocephalic trunk, LCC: left common carotid, LVA: left vertebral artery, LSA: left subclavian artery

Further examination of great vessels originating from the aortic arch revealed an unusual configuration of the great vessels. Generally, three great vessels, namely the brachiocephalic trunk, the left common carotid artery, and the LSA, originate from the aortic arch. However, an aberrant origin of the LVA from the aortic arch was noted just proximal to the origin of the LSA (Figure 1).

**Figure 2:**
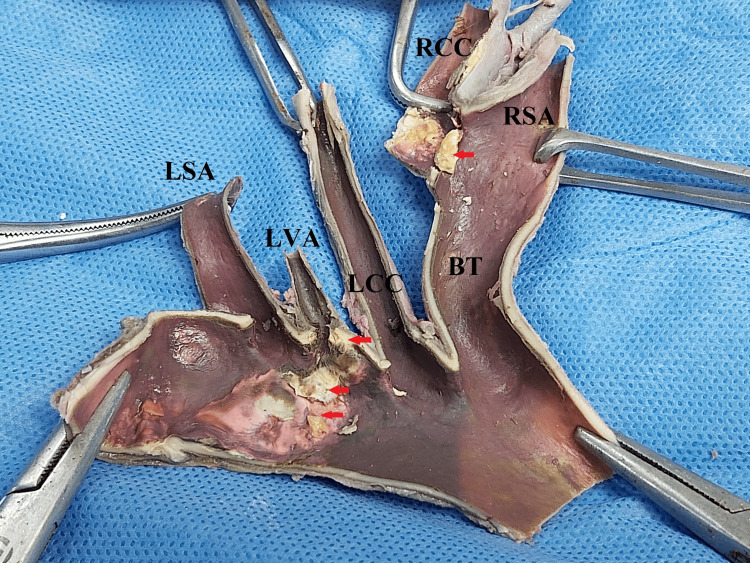
Branching pattern of vessels and atheromatous plaques near the origin of the right common carotid artery and LVA LSA: left subclavian artery, LVA: left vertebral artery, LCC: left common carotid, BT: brachiocephalic trunk, RCC: right common carotid, RSA: right subclavian artery

On further exploration, the LVA further ascends to its course by entering into the foramen of the transverse process in C4 vertebrae taking a longer course than usual. The wall of the aortic arch showed whitish-yellow atheromatous plaques on the cut section nearer to the origin of the right common carotid artery and LVA (Figure 2).

All other internal organs were intact and congested. Toxicological analysis of viscera showed negative results. The cause of death in the present case was given as sudden death caused by varying degrees of occlusion of the coronary arteries.

## Discussion

It is crucial to know the variation in the branching pattern of the aortic arch both from a clinical and forensic point of view. Such variation may be linked to aberrant development of aortic arches during embryonic life. Six aortic arches communicate between two dorsal aortas and the aortic sac. The first and second arches disappear when the remaining arches develop, forming a symmetric blood supply system during fetal life. Any variations in the fusion or absorption between the arches or aortic sac led to variation in a branching pattern. In the cervical region, small intersegmental branches of the descending aorta lose their connection with the aorta and anastomose to form the left and right vertebral arteries. Typically, the left seventh intersegmental branch gives rise to the LSA, which gives origin to the LVA. The persistence of this interconnection from the aortic arch results in the origin of the LVA from anomalous locations commonly along the axis of the aortic arch proximal to the left subclavian origin [2,4,5,7,9]. The variation of vertebral artery origin is more on the left side than the right and more unilateral than bilateral [7].

Adachi classified this variation into three types: A, B, and C. Type A variation is most common, observed in 80% of individuals. There are three major branches from the aortic arch: brachiocephalic trunk, left common carotid, and LSA. Type B variation is observed in 11% of individuals in which brachiocephalic and left common carotid arteries originate from the common trunk, not separately. A third variation, Type C, includes the origin of the LVA directly from the aortic arch proximal to the origin of the LSA [5].

The reported frequency of variation ranges between 0.68% and 5.8%. Vorster et al. [9] and Jayanthi et al. [4] noted that vertebral arteries originated directly from the aortic arch in 5% of cases. Williams and Edmonds [10] observed 407 American human cadavers and reported that an isolated LVA was noted in 2% of subjects. Anson and McVay [11] also observed this phenomenon in 2.5% of subjects. A study conducted by Patil et al. [5] on Indian cadaver subjects observed the isolated origin of the LVA from the aortic arch in 8% of subjects. They also suggested that the direct origin of the LVA from the aortic arch leads to increased blood flow to the brain on the left side, creating an imbalance between right and left circulation and may cause an increased incidence of cerebrovascular diseases in such patients [3,4,6].

Anomalous origin of the LVA usually does not result in clinical symptoms. Rarely, the patient may complain of dizziness without any identifiable connection to the aberrant origin of the vessel [7]. However, no gross abnormal cerebrovascular finding was observed in the present case; the patient presented with a history of chest pain followed by unresponsiveness. The heart's internal results and vasculature, such as white patches on the epicardium, the atheromatous blockade in the coronaries, and the anomalous origin of the LVA, were linked with the deceased's death.

The origin of the LVA directly from the aortic arch makes it prone to develop atheromatous plaques due to high blood turbulence flow. According to Gluncic et al. [12], the vertebral artery's prevertebral segment is frequently affected by atherosclerosis. According to Phan et al., the patients may be asymptomatic unless atherosclerotic lesions involve the vertebral artery [13].

Yuan [8] conducted a study in a cohort of 1286 cases involving 955 patients and 331 cadavers. The results indicate more left than right and more unilateral than bilateral aberrant vertebral artery origin. Patients with anomalous vertebral artery origin were often asymptomatic, and only 5.5% of the patients were symptomatic, probably due to the vertebral artery's anomalous origin.

Vascular variation can cause changes in blood flow and, thus, may increase the chances of aneurysm formation in the vertebrobasilar system of the body [14]. For the same reason, special attention should be taken to surgeons dealing with spinal surgeries. The vertebrobasilar system should be handled carefully to prevent massive bleeding in the patient [14,15]. Vertebrobasilar artery insufficiency can have an 80-95% mortality rate in a patient [16]. Computed tomography of the vasculature may be suggested before surgery whenever there is suspicion of an anomalous vertebral artery [17]. Komiyama et al. reported a higher incidence of arterial dissection in the LVA originating directly from the aortic arch in comparison to their normal anatomical origin [18].

In live patients, the diagnosis of an aberrant vertebral artery can easily be made out with color Doppler. In addition, other modalities can achieve cerebrovascular imaging, such as chest and neck vessels' angiography or magnetic resonance imaging and computed tomography angiography [15,17]. During postmortem examination, careful dissection of the vascular system is warranted especially in case of sudden death. Various postmortem imaging modalities might prove helpful in this regard. Gross examination supported by imaging methods definitely helps to identify any anomalous origin of great vessels from the aortic arch, thus helping correlate with the cause of death if any.

## Conclusions

The vertebral arteries originate from the subclavian arteries on both sides. However, the uncommon origin of the left vertebral arteries has been documented in the literature. In such a scenario, the LVA may originate directly from the aortic arch instead of the LSA. These anomalous origins are not always a cause of concern for the patient. However, sometimes it can cause changes in cerebral hemodynamics, leading to cerebrovascular complications.

Knowing the anatomical and morphological variation of the vertebral artery is crucial in clinical settings because it might affect the prognosis of the patient. The medical professional should act cautiously while performing specific medical procedures including angiograms or surgeries of the head and neck regions for taking necessary action. During postmortem procedures also, it is imperative to identify such anomalies by careful dissection of the vasculature while assessing its relation with the cause of death of the patient. It may be just an incidental finding during the autopsy, but such anomalies are always a significant learning value in the field of forensic medicine.
